# External validation of a minimal-resource model to predict reduced estimated glomerular filtration rate in people with type 2 diabetes without diagnosis of chronic kidney disease in Mexico: a comparison between country-level and regional performance

**DOI:** 10.3389/fendo.2024.1253492

**Published:** 2024-03-22

**Authors:** Camilla Sammut-Powell, Rose Sisk, Ruben Silva-Tinoco, Gustavo de la Pena, Paloma Almeda-Valdes, Sonia Citlali Juarez Comboni, Susana Goncalves, Rory Cameron

**Affiliations:** ^1^ Gendius Ltd, Alderley Edge, United Kingdom; ^2^ Clinic Specialized in the Diabetes Management of the Mexico City Government, Public Health Services of the Mexico City Government, Mexico, City, Mexico; ^3^ Department of Endocrinology and Metabolism, Instituto Nacional de Ciencias Médicas y Nutrición Salvador Zubirán (INCMNSZ), Mexico City, Mexico; ^4^ Metabolic Diseases Research, Instituto Nacional de Ciencias Médicas y Nutrición Salvador Zubirán, Mexico City, Mexico; ^5^ AstraZeneca, Mexico City, Mexico; ^6^ International Region, AstraZeneca, Buenos Aires, Argentina

**Keywords:** type 2 diabetes, chronic kidney disease, screening, risk stratification, clinical prediction model, low-and-middle-income countries

## Abstract

**Background:**

Patients with type 2 diabetes are at an increased risk of chronic kidney disease (CKD) hence it is recommended that they receive annual CKD screening. The huge burden of diabetes in Mexico and limited screening resource mean that CKD screening is underperformed. Consequently, patients often have a late diagnosis of CKD. A regional minimal-resource model to support risk-tailored CKD screening in patients with type 2 diabetes has been developed and globally validated. However, population heath and care services between countries within a region are expected to differ. The aim of this study was to evaluate the performance of the model within Mexico and compare this with the performance demonstrated within the Americas in the global validation.

**Methods:**

We performed a retrospective observational study with data from primary care (Clinic Specialized in Diabetes Management in Mexico City), tertiary care (Instituto Nacional de Ciencias Médicas y Nutrición Salvador Zubirán) and the Mexican national survey of health and nutrition (ENSANUT-MC 2016). We applied the minimal-resource model across the datasets and evaluated model performance metrics, with the primary interest in the sensitivity and increase in the positive predictive value (PPV) compared to a screen-everyone approach.

**Results:**

The model was evaluated on 2510 patients from Mexico (primary care: 1358, tertiary care: 735, ENSANUT-MC: 417). Across the Mexico data, the sensitivity was 0.730 (95% CI: 0.689 – 0.779) and the relative increase in PPV was 61.0% (95% CI: 52.1% - 70.8%). These were not statistically different to the regional performance metrics for the Americas (sensitivity: p=0.964; relative improvement: p=0.132), however considerable variability was observed across the data sources.

**Conclusion:**

The minimal-resource model performs consistently in a representative Mexican population sample compared with the Americas regional performance. In primary care settings where screening is underperformed and access to laboratory testing is limited, the model can act as a risk-tailored CKD screening solution, directing screening resources to patients who are at highest risk.

## 1 Introduction

Diabetes is a leading cause of kidney disease and with the rising prevalence of type 2 diabetes (1), the burden of chronic kidney disease (CKD) is also expected to increase (2). The burden of CKD in Mexico is growing at an exponential rate (3), yet, the majority of patients with CKD do not receive a timely diagnosis nor treatment (4). The Kidney Early Evaluation Program (KEEP) Mexico currently supports the early detection of CKD amongst adults with risk factors, such as diabetes, but its outreach remains low (8858 individuals screened between 2008 and 2017) (5). Therefore, a targeted approach to screening that can be implemented within health care services may offer a larger-scale solution to the early detection of CKD.

A minimal resource model to predict a reduced estimated glomerular filtration rate (eGFR below 60 ml/min/1.73m^2^) in patients with type 2 diabetes has been previously developed and globally validated (6–8). The model uses age, sex, duration of diabetes, body mass index and blood pressure to predict the probability that the patient’s eGFR is below 60, potentially indicating undiagnosed CKD. If the probability is 11.5% or higher, the patient is deemed ‘high risk’ and it is recommended that the patient is prioritized for CKD screening. This threshold was selected to achieve on average a sensitivity of 80%, whilst simultaneously improving the positive predictive value (PPV) compared to a screen-everyone approach, where only 10-20% have an eGFR below 60. The purpose of the model is to support earlier identification of CKD by understanding which patients are at highest risk and using this information to strategically allocate CKD screening resource to those most in need. The global validation used AstraZeneca’s global registry data (iCaReMe and DISCOVER) and demonstrated promising potential to refine the population to those at highest risk, whilst retaining a high detection rate: overall, a relative improvement of 50% was observed in the PPV, whilst retaining a sensitivity of 80%. However, the validation focused on global and regional performance, using the World Health Organization (WHO) classification of region. Most contributing countries had small samples; for the Americas, the total sample size was 1430 patients made up from 7 countries (Canada and Latin America), each contributing between 28 and 296 patients. Therefore, the results may not be generalizable to individual country-level performance.

Healthcare systems and patient populations vary considerably across Latin America (9). For example, in Brazil the population is made up of a higher proportion of Afro-descendants, whereas Mexico has a higher proportion of indigenous people, and the Mexican population was shown to be three times more likely to present with complications of diabetes (10). Consequently, the regional performance may not be representative of its performance within Mexico. Therefore, we aimed to perform a country-level validation of the minimal-resource model in Mexico and compare it against the regional performance for the Americas.

## 2 Materials and methods

### 2.1 Design and data sources

This was a retrospective, observational study using data collected from: the DIABEMPIC programme within the Clinic Specialized in Diabetes Management in Mexico City, consisting of data collected from patients with continuous medical care in 32 public primary care units between 2^nd^ January 2017 and 8^th^ December 2022; Instituto Nacional de Ciencias Médicas y Nutrición Salvador Zubirán (INCMNSZ), consisting of data from patients attending a tertiary care center for specialized care, located in Mexico City, collected between 20^th^ September 2020 and 10^th^ November 2022; and the Encuesta Nacional de SAlud y NUTricion 2016 (ENSANUT-MC 2016), i.e. the Mexican National Survey of Health and Nutrition (11).

Ethical approval was obtained on a clinic-by-clinic basis, adhering to policy within each clinic. All adults (aged 18 and over) with a diagnosis of type 2 diabetes were included. Patients with a disease diagnosis code (e.g. ICD-10 code) of CKD stage 3-5 were excluded.

### 2.2 Prediction model

We implemented the minimal-resource CKD pre-screening model developed by Gendius, described in Sammut-Powell et al. (6). The model uses age, gender, body mass index, time since diagnosis of diabetes and blood pressure to predict the probability that a patient’s eGFR is below 60 ml/min/1.73m^2^, herein referred to as the predicted probability. A pre-determined threshold of 11.5% (derived during model development) was used to categorize patients as high risk or not, i.e. if the risk was 11.5% or higher, the patient was categorized as ‘high risk’.

### 2.3 Primary outcome

The primary outcome was an indicator of whether the eGFR was below 60 ml/min/1.73m^2^ or not, consistent with the definition in the model. The eGFRs were provided directly from the clinics. However, for the survey data, only the serum creatinine was available, therefore we calculated the eGFR using the 2009 CKD-EPI formula (12).

### 2.4 Missing data

All demographic data for patients with corresponding outcome data were complete.

### 2.5 Statistical methods

Population summary statistics were evaluated to compare the populations within the clinics. T-tests and Wilcoxon rank-sum tests were performed to determine statistical differences between patient demographics that had a normal and non-normal distribution, respectively.

#### 2.5.1 Model performance

The model was applied to the data and the distribution of the predicted risks were visualized by data source. The sensitivity and the relative improvement in the positive predictive value (PPV) were the primary performance metrics. Relative improvement in the PPV is defined as the improvement over a screen-everyone approach, where the PPV of a screen-everyone approach is equal to the prevalence of eGFR below 60 ml/min/1.73m^2^.

Secondary performance metrics included the specificity, negative predictive value, and the C-statistic. Binomial regression and Poisson tests were used to compare the sensitivity and relative improvement in PPV estimates, respectively, with the estimates previously obtained during the global registry validation for the Americas. The 95% bootstrap confidence intervals (CI) were obtained using 200 samples. Calibration was assessed visually.

#### 2.5.2 Sample size requirements

A total of 220 positive cases from the Americas were included in global validation. Consequently, an additional 321 positive cases from Mexico corresponds to an 80% power for a 5% significance level to detect a reduction of 10% in the sensitivity, using a Normal approximation and the sensitivity estimate of 0.732 for the Americas, as previously observed.

All analyses were performed in R.

## 3 Results

**Figure 1:**
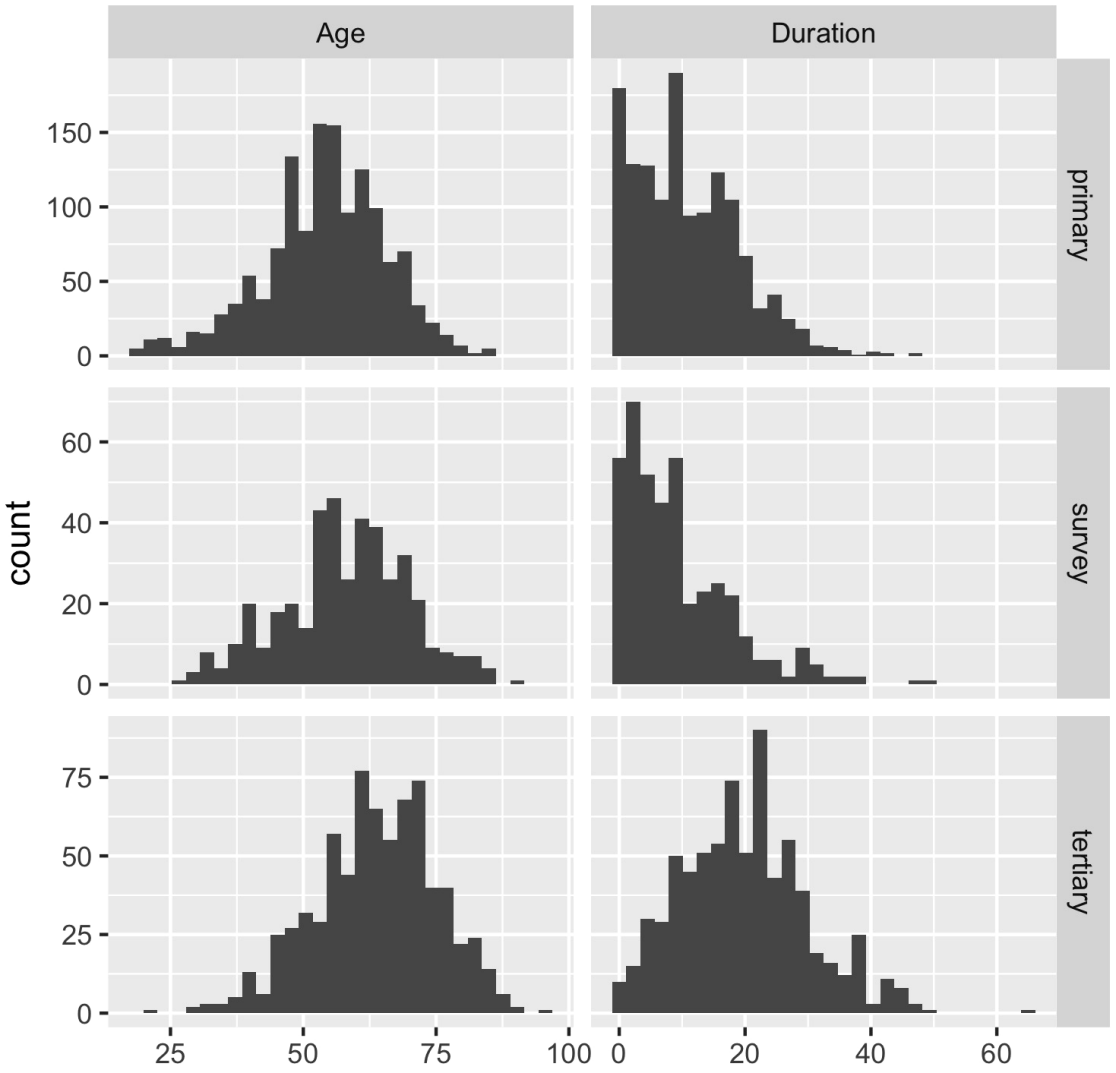
Histograms of the age of the patients and duration (time since diagnosis) of type 2 diabetes in years across the Mexican data sources (primary = primary care clinics; tertiary = tertiary care clinic; survey = ENSANUT-MC 2016).

There were 2510 patients identified as aged 18 or above, diagnosed with type 2 diabetes and no previous diagnosis of CKD stage 3-5, with a serum creatinine or eGFR and no missing data (primary care: 1358, tertiary care: 735, ENSANUT-MC: 417). The prevalence of an eGFR below 60ml/min/1.73m^2^ was 11.7% and 20.1%, in the primary and tertiary clinics, respectively (**Table 1**). The tertiary clinic population consisted of older patients with a longer duration of type 2 diabetes, compared to the primary care (age: p<0.001, duration: p<0.001) and survey (age: p<0.001, duration: p<0.001) populations (**Figure 1**). Overall, the Mexico population sample was statistically significantly different across the majority of demographics but not clinically different in age and BMI.

**Table 1:** Baseline demographics of patients across primary and tertiary care services in Mexico City and a representative population sample from a nutritional survey (ENSANUT-MC).

	Americas, Global Validation (registry)	Mexico Validation				
		Total		Primary Clinics	ENSANUT-MC (survey)	Tertiary Clinic
	n = 1430	n = 2510	p-value	n = 1358	n = 417	n = 735
Gender, n (%)						
Female	685 (47.9%)	1390 (55.4%)	<0.001	825 (60.8%)	117 (28.1%)	448 (61%)
Male	745 (52.1%)	1120 (44.6%)		533 (39.2%)	300 (71.9%)	287 (39%)
Age (years), mean (SD)	58.7 (12.1)	57.6 (12.3)	0.009	54.1 (11.6)	58.1 (11.9)	63.8 (11.5)
Body mass index (kg/m^2^), mean (SD)	30.6 (5.7)	29.2 (5.8)	<0.001	29.7 (6.0)	29.7 (5.6)	28.1 (5.1)
Time since diagnosis of diabetes (years), median (LQ-UQ)	5.2 (2.2-10.4)	12 (5-20)	<0.001	10 (4-16)	7 (3-14)	20 (13-26)
Diastolic blood pressure (mmHg), mean (SD)	79.3 (10.9)	74.2 (10.4)	<0.001	74.3 (10.3)	74.7 (11.7)	73.5 (9.5)
Systolic blood pressure (mmHg), mean (SD)	130.7 (17.1)	125.3 (19.6)	<0.001	124.7 (20.0)	131.4 (22.1)	122.9 (16.6)
eGFR (ml/min/1.73m^2^), median (LQ-UQ)	87.5 (69.9-101.2)	93.5 (73-105.5)	<0.001	94.2 (76.6-104.9)	103.5 (85.4-118)	89 (63-99)
eGFR below 60, n (%)	220 (15.4%)	352 (14%)	0.263	159 (11.7%)	45 (10.8%)	148 (20.1%)
CKD G-stage, n (%)						
Stage G0-1	664 (46.4%)	1433 (57.1%)	<0.001	779 (57.4%)	293 (70.3%)	361 (49.1%)
Stage G2	546 (38.2%)	725 (28.9%)		420 (30.9%)	79 (18.9%)	226 (30.7%)
Stage G3	174 (12.2%)	304 (12.1%)		151 (11.1%)	39 (9.4%)	114 (15.5%)
Stage G4-5	46 (3.2%)	48 (1.9%)		8 (0.6%)	6 (1.4%)	34 (4.6%)

Statistical tests between the Americas population and combined Mexico population data were performed to determine statistical differences in the populations; a p-value of below 0.05 was considered significant. SD, standard deviation; LQ, lower quartile; UQ, upper quartile; eGFR, estimated glomerular filtration rate.

**Figure 2:**
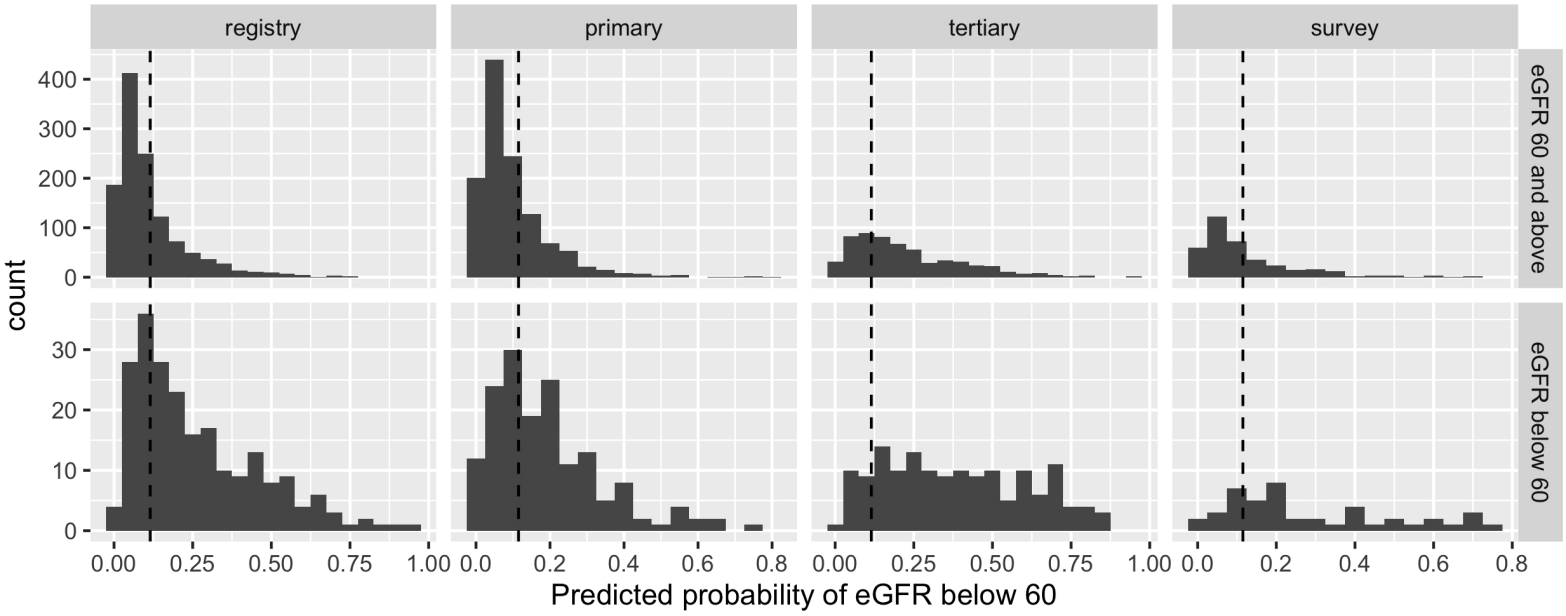
Histograms of predicted probabilities of eGFR below 60ml/min/1.73m^2^ by data source (registry = Americas, Global Validation; primary = primary care clinics; tertiary = tertiary care clinics; survey = ENSANUT-MC 2016) and observed eGFR below 60ml/min/1.73m^2^ or not. The cut-off for categorising a patient as high risk is indicated by a dashed line; probabilities to the right of the line are high risk.

The overall sensitivity averaged across all the data from Mexico (0.730, 95% CI: 0.689 – 0.779) was not statistically different to that previously reported for the Americas in a global registry validation (p=0.964). However, considerable variability was observed in the sensitivity estimates across the data sources within Mexico (Primary clinics: 0.592; ENSANUT-MC survey: 0.733; Tertiary clinic: 0.872; **Table 2**). No statistical difference was observed between the sensitivity observed in the survey data and the Americas registry data, but the sensitivity was statistically lower in the primary clinics (p=0.006) and higher in the tertiary clinic (p=0.001) compared to the sensitivity obtained for the Americas (**Figure 2**).

**Table 2:** Estimates and 95% bootstrap confidence intervals of performance metrics.

	Global Validation (Americas)	Mexico Validation				
		Total		Primary Clinics	ENSANUT-MC (survey)	Tertiary Clinic
	n = 1430	n = 2510	p-value	n = 1358	n = 417	n = 735
Prevalence	0.154 (0.136 - 0.173)	0.140 (0.127 - 0.155)	0.263	0.117 (0.099 - 0.134)	0.108 (0.079 - 0.132)	0.201 (0.175 - 0.226)
Positive Predictive Value (PPV)	0.288 (0.256 - 0.326)	0.226 (0.204 - 0.252)	0.005	0.209 (0.171 - 0.245)	0.214 (0.164 - 0.277)	0.244 (0.211 - 0.287)
Relative improvement in PPV	0.872 (0.735 - 1.052)	0.610 (0.521 - 0.708)	0.132	0.783 (0.543 - 1.006)	0.986 (0.703 - 1.394)	0.211 (0.144 - 0.284)
Sensitivity	0.732 (0.683 - 0.792)	0.730 (0.689 - 0.779)	0.964	0.597 (0.520 - 0.667)	0.733 (0.622 - 0.865)	0.872 (0.820 - 0.926)
Specificity	0.671 (0.646 - 0.698)	0.592 (0.573 - 0.613)	<0.001	0.700 (0.674 - 0.727)	0.675 (0.628 - 0.723)	0.319 (0.289 - 0.361)
Negative Predictive Value (NPV)	0.932 (0.915 - 0.950)	0.931 (0.918 - 0.943)	0.891	0.929 (0.912 - 0.943)	0.954 (0.932 - 0.981)	0.908 (0.869 - 0.945)
C-statistic	0.768 (0.738 - 0.805)	0.733 (0.711 - 0.761)	0.113	0.718 (0.675 - 0.760)	0.779 (0.722 - 0.848)	0.706 (0.659 - 0.750)
Proportion predicted high risk	0.391 (0.367 - 0.417)	0.453 (0.436 - 0.474)	<0.001	0.335 (0.311 - 0.359)	0.369 (0.328 - 0.415)	0.720 (0.687 - 0.750)

P-values to test for statistical differences between the Americas population sample and the Mexico population sample are presented.

For the primary care and survey populations, most patients with an eGFR of 60 ml/min/1.73m^2^ and above had a predicted probability that was below the cut-off (**Figure 2**); the specificity remained between 0.671 and 0.700 within the regional, primary care and survey populations. However, it was significantly reduced in the tertiary care clinic, evidencing the trade-off from achieving a high sensitivity. Despite this, the negative predictive value remained above 0.9 across all populations, likely due to the low prevalence of patients with an eGFR below 60 ml/min/1.73m^2^.

The average relative improvement in the PPV was not statistically different in the Mexico data compared to that previously demonstrated for the Americas (p=0.132), despite the estimate being heavily discounted by the performance in the tertiary care clinic whose individual performance was statistically lower (p<0.001). The primary care and survey data relative improvements in PPV were not statistically different to that estimated for the Americas (primary clinic: p=0.748; survey: p=0.770).

**Figure 3:**
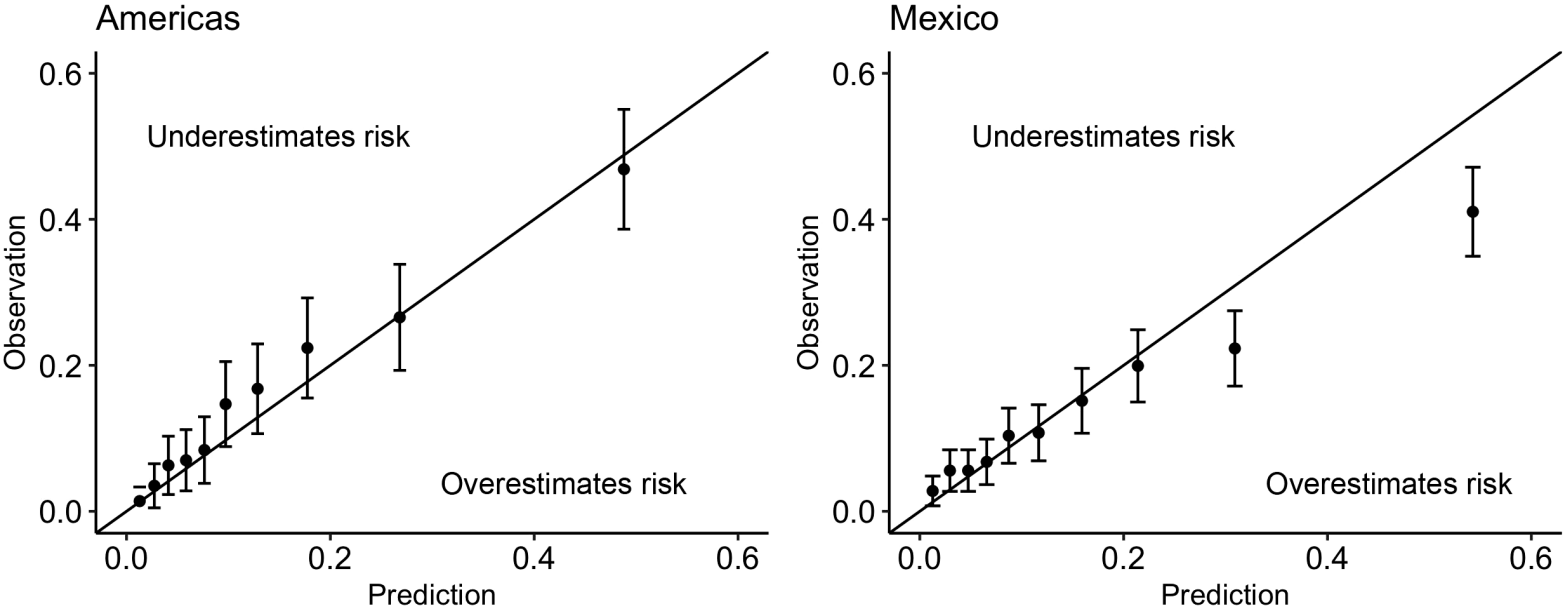
Calibration plots for the minimal-resource model when applied to (left): the Americas registry data used in the Global Validation and (right): the combined primary care, tertiary care and survey data from Mexico. Above the line indicates that the risk was underestimated by the model; below the line indicates that the risk was overestimated by the model.

The discrimination was good (C-statistic above 0.7) across all data sources (**Table 2**). The model remained well-calibrated in the Mexico data within those with a predicted probability below 0.2 (**Figure 3**). For larger probabilities, the model was observed to overestimate the risk. For both the primary care and survey populations, the proportion of patients predicted as high risk was below 40%, indicating a large reduction in the population by selecting only patients that are high risk.

## 4 Discussion

We have demonstrated that the regional performance was consistent with the average performance using a large sample from Mexico; the minimal-resource model reduced the screening population to significantly increase the positive predictive value, whilst retaining an adequate sensitivity. However, performance across data sources varied. Therefore, care must be taken when anticipating how the model will perform in various clinical settings, and establishing whether use of the model in a particular setting is appropriate or supported by sufficient performance estimates.

Differences in populations and performance estimates between the care services can be partly explained by the care system in Mexico. Unlike other healthcare services such as UK primary care, the primary care service in Mexico does not serve all patients. Instead, the population is split across primary, secondary, and tertiary care. Therefore, data from a single service are not representative of the whole population. To get an overall estimate of the performance across the population, population study data or a collection of data from all care services with a population weighting is required. Therefore, comparing performances between the regional and the survey data is likely to attenuate selection bias. Additionally, the Americas population primarily consisted of patients from the DISCOVER study (n=933/1430) which enrolled patients at initiation of second-line glucose-lowering therapy and therefore may explain differences in baseline demographics and performance metrics within single-service populations.

Whilst the sensitivity of the model was below the desirable threshold in the primary care clinics, the sensitivity within the tertiary care clinic surpassed the minimum required performance. Therefore, the model appears to be more conservative to ruling-out patients that may have a worse overall health, likely due to confounding with age. This aligns with current clinical practice to remain risk-averse in comorbid patients with severe health conditions; in tertiary clinics, risk factor monitoring of patients is routinely performed across all patients, regardless of symptoms. The inherent trade-off between sensitivity and specificity was evident, with a much lower specificity observed within the tertiary care clinic. Therefore, the application of the model may be most beneficial in primary and secondary care services where screening is not routinely performed. When adopting only within these services, expectations of the model’s performance should be adjusted to accommodate the change in the overarching population.

The Mexican National Health and Nutritional Survey data has been widely used amongst researchers and is considered to be a representative sample of the Mexican population (13–15), however it is difficult to assert conclusively that the samples of patients in the primary and tertiary clinics are representative of the entire country, particularly because they are from an urban population. Therefore, the survey data provides a more comparable population performance within Mexico. The performance within this subgroup was most similar to the performance previously estimated for the Americas region. However, the survey data is subject to limitations. Firstly, the sample size was small, meaning that these estimates may not be reliable. Secondly, the only way to identify a previous CKD diagnosis was through a patient self-reporting that they had been told they have kidney failure. In contrast, the clinics were able to determine whether a patient has a CKD diagnosis code and therefore the patient selection within the survey population may not be consistent with the clinic data. Thirdly, the data are taken from 2016 and therefore may not be representative of the current population, given temporal changes to population health and care practices.

Intensified multifactorial interventions to control major risk factors, e.g., HbA1c, blood pressure, and lipids, are associated with a reduced risk of CKD incidence or progression and other relevant outcomes in patients with type 2 diabetes mellitus (16, 17). Moreover, clinical trials have demonstrated the effectiveness of the sodium-glucose cotransporter inhibitor 2 (SGLT2i) in delaying progression of CKD (18–20), yet prescribing remains low (21). Given the significant burden of CKD within Mexico (3), it is imperative that there is a strategy to identify and treat patients in a timely fashion. Using the minimal-resource model provides a solution to support identification of high-risk patients enabling targeted screening programs with higher efficiency than standard practice, especially where standard practice is sub-par due to limited available resources. For example, in primary care, patients that are identified as high risk for undiagnosed CKD may be targeted to receive a diagnosis assessment and in those with confirmatory results, a patient may be transitioned to secondary or tertiary care, and new treatments may be initiated to slow the progression of the disease.

Mexico has the world’s sixth-highest premature death rate from CKD (22). From 1990 to 2017, the country’s age-standardized CKD mortality rate jumped from 28.7 to 58.1 per 100,000 inhabitants (3, 22). This jump represented an increase in the mortality rate of 102.3%, while the increases in the rates of Latin America and the worldwide population were 32.9 and 2.8%, respectively (22, 23). This remarkable increase in the mortality rate is explained by a growing burden of risk factors such as diabetes, restricted access to preventive interventions and resources supporting early management of the disease, and limited availability of renal replacement therapy, primarily among the low-income population (3, 22, 24).

In low- and middle-income countries, like Mexico, barriers to provision and access to diabetes care are compounded by the often-fragmented care pathways for the multiple needs of many people with diabetes. These impact patients’ adherence to treatment, diabetes care goals attainment, and the screening of complications (25–27). For example, patients without health insurance (mainly unsalaried workers, the unemployed and the economically inactive population) have no guarantee of adequate medical follow-up and screening for complications related to diabetes (25). As a result, the differences in coverage and access to medical care for patients with CKD have greatly contributed to deepening the health inequities among the Mexican population, with the most unfavourable results occurring among its poorest members.

In Mexico, as in other low- and middle-income countries, routine procedures to detect and diagnose CKD are not sufficiently performed (28, 29). This has resulted in a high rate of undiagnosed and undertreated patients (30). Given that CKD progresses slowly, without pain or discomfort, and irreversibly, patients may not know that they have it for years, especially at primary and secondary levels of the care system. Many patients spend time presenting chronic poor metabolic control with silent deterioration of renal function until very advanced stages, where the only viable treatment option is renal replacement therapy. This illustrates the importance of strengthening primary level care and improving early detection of CKD, particularly for those at high-risk (those who are overweight or obese or have diabetes or hypertension).

Alternative published models to predict diabetic kidney disease are largely prognostic (31–36), i.e. remain focused on predicting future onset of disease as opposed to undiagnosed CKD, or are not specific to the population with type 2 diabetes (15, 37). They often include measurements from invasive testing (33, 38) which may not be available in low-to-middle income countries and therefore are unsuitable for use in such settings. The minimal-resource model removes barriers to application through requiring only readily available information. It can be utilised during a patient consultation or within community care to instantly assess a patient’s risk. Where electronic health records are available, the model can be applied using the most recent measurements to risk-stratify the entire patient group. Upon identifying a patient as high risk, a clinician can prioritize the patient for CKD screening and act with urgency, supporting early detection.

## 5 Conclusions

The minimal-resource model performs consistently in a representative Mexican population sample compared with the Americas regional performance using registry data. When applying the model within an individual clinic or sector of healthcare, the performance is expected to vary, aligned with the health of the population served by the clinic. In primary care settings where screening is underperformed and access to laboratory testing is limited, the model can act as a risk-tailored CKD screening solution, directing screening resources to patients who are at highest risk. This may enable earlier detection of CKD and an opportunity to intervene at a time when the course of the disease may be changed.

## Data availability statement

The primary care and tertiary care datasets presented in this article are not readily available because the patients have not given written consent to share their data publicly. Requests to access the datasets should be directed to ruben_ost@hotmail.com and paloma.almedav@incmnsz.mx respectively. Data from the ENSANUT-MC 2016 are freely available from https://ensanut.insp.mx.

## Ethics statement

Ethical approval was not required for the study involving humans in accordance with the local legislation and institutional requirements. Written informed consent to participate in this study was not required from the participants or the participants’ legal guardians/next of kin in accordance with the national legislation and the institutional requirements.

## Author contributions

CS-P: Conceptualization, Formal analysis, Methodology, Visualization, Writing – original draft, Writing – review & editing. RS: Conceptualization, Writing – review & editing. RS-T: Data curation, Writing – original draft, Writing – review & editing. GD: Data curation, Writing – original draft, Writing – review & editing. PA-V: Data curation, Writing – original draft, Writing – review & editing. SJ: Conceptualization, Writing – review & editing. SG: Conceptualization, Writing – review & editing. RC: Conceptualization, Writing – review & editing.
